# Comparison of Dynamics of Udder Skin Microbiota From Grazing Yak and Cattle During the Perinatal Period on the Qinghai–Tibetan Plateau

**DOI:** 10.3389/fvets.2022.864057

**Published:** 2022-05-27

**Authors:** Jianbo Zhang, Anum Ali Ahmad, Yan Jia, Renqing Dingkao, Mei Du, Zeyi Liang, Juanshan Zheng, Ibrahim Bature, Ping Yan, Ghasem Hosseini Salekdeh, Xuezhi Ding

**Affiliations:** ^1^Key Laboratory of Yak Breeding Engineering, Lanzhou Institute of Husbandry and Pharmaceutical Sciences, Chinese Academy of Agricultural Sciences, Lanzhou, China; ^2^Key Laboratory of Veterinary Pharmaceutical Development, Ministry of Agricultural and Rural Affairs, Lanzhou Institute of Husbandry and Pharmaceutical Sciences, Chinese Academy of Agricultural Sciences, Lanzhou, China; ^3^Gannan Institute of Animal Husbandry Science, Hezuo, China; ^4^Department of Systems Biology, Agricultural Biotechnology Research Institute of Iran, Agricultural Research, Education, and Extension Organization, Karaj, Iran

**Keywords:** udder skin microbiota, core microbiota, perinatal period, postpartum, yak, cattle

## Abstract

The perinatal period has an important impact on the health of ruminants, and the imbalance of udder skin microbiota might be an important inducement of bovine mastitis. However, it is not clear how the perinatal period affects the microbial structure and stability of the udder skin of yak and cattle. Here, we used 16S rRNA gene high-throughput sequencing to analyze the udder skin microbiota of yak and cattle during the perinatal period. We found that the diversity and richness of microbiota of bovine udder skin during 1–2 weeks postpartum were significantly lower than those in the 1–2 weeks prenatal and 1-month postpartum period (Wilcoxon, *p* < 0.05). Besides, we found sharing of 2,533 OTUs in the udder skin microbiota of yak and cattle during the perinatal period, among which the core microbiota at the genera level was mainly composed of *Staphylococcus, Moraxella*, and *Acinetobacter*. However, the genus *Acinetobacter* was significantly abundant in the udder skin of cattle during 1–2 weeks postpartum. The NMDS and LEfSe results showed that the perinatal period had more effects on the composition and stability of microbial community in the udder skin of cattle compared to yak, particularly during 1–2 weeks postpartum. In addition, the average content of total whey proteins and immunoglobulin G of whey protein were significantly higher in the yak colostrum when compared to those found in the cattle (*p* < 0.05). In conclusion, the structure of udder skin microbiota of yak during the perinatal period is more stable than that of cattle in the same habitat, and 1–2 weeks postpartum may be a potential window period to prevent cattle mastitis.

## Introduction

The skin, functioning as an external interface between the body and the environment, acts as a physical barrier to prevent the invasion of foreign pathogens while providing a habitat for the commensal microbiota (1, 2). Studies have shown that this commensal microbiota, including bacteria, fungi, viruses, and mites, has the potential to contribute to the alteration of the skin immune function (3). The skin is constantly exposed to environmental bacteria that can become transient and resident members of the host community, some of which are potentially pathogenic (2). Once the balance of this commensal microbiota is disturbed, it may lead to skin infections or diseases, such as cow mastitis (4). Studies conducted to date have suggested that an optimum diversity of mammary microbiota is associated with immune homeostasis, whereas the microbiota of mastitis quarters, or those with a history of mastitis, are considerably less diverse (5). Therefore, a healthy and stable commensal skin microbiota plays an important role in both influencing the skin immune response and acting as a barrier against colonization of potentially pathogenic microorganisms and overgrowth of opportunistic pathogens.

The Qinghai–Tibetan Plateau (QTP) offers one of the most extreme environments (i.e., high altitude, hypoxia, long cold season, and strong ultraviolet radiation) for the survival of human and other mammalian species (6). The yak (*Bos grunniens*), a herbivore exclusively inhabiting the QTP and adjacent high-altitude regions, was differentiated from cattle (*Bos taurus*) about 4.4–5.3 million years ago (7). After long periods of natural selection and evolution, yak were found to be superior to cattle in feeding and grazing behavior (8), digestive organ structure (9, 10), nitrogen use efficiency (11), low rumen methane emission (12), and interseason energy utilization efficiency (13, 14). In addition, yak milk is considered to be a natural concentrated dairy product and is an important part of the daily diet of Tibetan nomads. Compared with dairy cow milk, the fat content of yak milk [range of 5.3–8.8% (*w*/*v*)] is almost twice that of dairy cow (*Bos taurus*) milk (15), but its yield is only about 10% of dairy cow milk (6). Compared to some autochthonous cattle breeds, high-yielding dairy cattle may be more prone to metabolic stress and mastitis (16, 17). At present, it is not clear how the perinatal period affects the structure and stability of the udder skin microbiota of grazing yak and cattle on the QTP. However, yak have adapted to the extreme natural environment of the QTP over long periods of evolution, and we hypothesized that the structure and function of the udder skin microbiota of healthy yak during the perinatal period were more stable compared to cattle inhabiting the same habitat. Therefore, based on the 16S rRNA gene amplification sequence, we described and compared the structure and succession of udder skin microbiota of healthy yak and cattle during the perinatal period under the same management conditions, and the core microbiota of udder skin was screened. The aim of this study was to describe the changes in udder skin microbiota and its functional potential in the healthy cattle and yak from Qinghai–Tibetan Plateau during the perinatal period.

## Materials and Methods

### Animal Management, Experiments, and Sampling

All the animals involved in this experiment were from the same herd, and they grazed together on the same native pasture (without any supplementation) of Yangnuo Specialized Yak Breeding Cooperative (34°43'19.66“N, 102°28'49.51”E) at Xiahe County of Gannan Tibetan Autonomous Prefecture, Gansu Province, China. In this study, yak and cattle were naturally mated and delivered, and the calves were suckled by the dam in the same pasture. To ensure that all the animals remained clinically healthy throughout the study duration, we had to have a veterinarian check the clinical phenotype of bovine mastitis before each sampling to ensure the health of yak and cattle. Unfortunately, we have not been able to verify the udder health of the test animals through specific laboratory tests.

**Figure 1:**
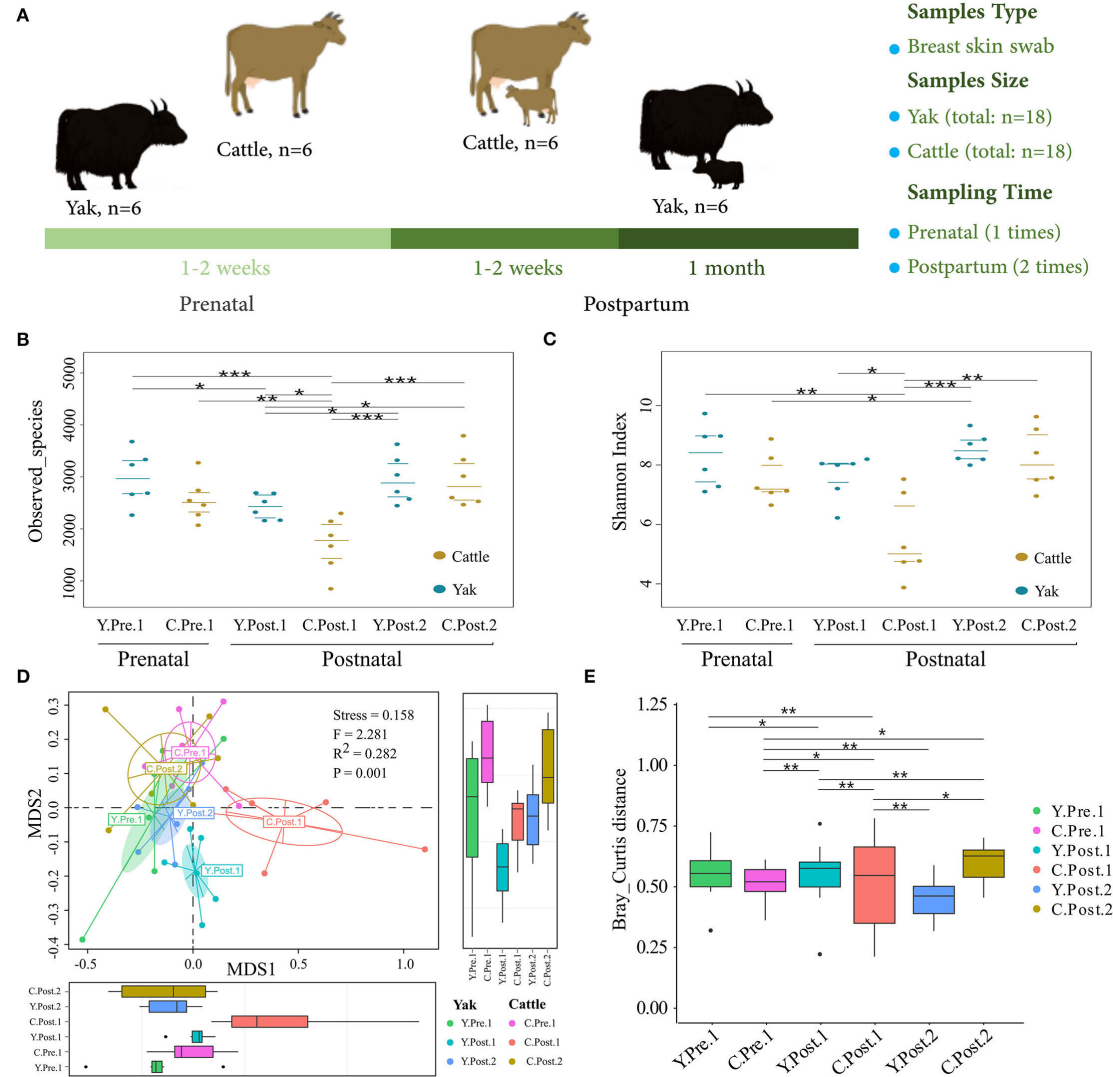
Abundance and diversity of udder skin microbiota of yak and cattle during the perinatal period. **(A)** Experimental design, including sample collection time, sample type, and sample number. The diversity and richness of microbial communities in the udder skin of yak and cattle in different periods during the perinatal period were analyzed. The observed species richness **(B)** and Shannon Diversity Index **(C)** are shown by beeswarm plots. **(D)** Based on Bray_Curtis distance, NMDS maps of the microbial community in the udder skin of yak and cattle were drawn, and the udder skin microbiota in different periods during the perinatal period were examined by PERMANOVA. **(E)** The box plots were used to show the differences in the microbial communities in the udder skin of yak and cattle during different periods of the perinatal period. The difference between two groups was considered statistically significant when the *p*-value was < 0.05 (*p < 0.05; **p < 0.01; ***p < 0.001).

Six female yaks (BW = 258.3 ± 22.5 kg) and six female Tibetan cattle (BW = 246.5 ± 16.5 kg), whose age ranged between 4 and 6 years, from the same herd were selected and freely grazed on natural alpine meadow herbage without any supplementary feed from 7 a.m. to 6 p.m. with free access to water from the local river. Udder skin swabs from yak and cattle were obtained by swabbing udder skin (teat apex, teat barrel, and base) with a sterile cotton swab during the perinatal period. An overview of the experimental design is shown in Figure 1A. Udder skin samples from yak and cattle were collected repeatedly before grazing in the morning for 1–2 weeks prenatal (Y.Pre.1: *n* = 6; C.Pre.1: *n* = 6), 1–2 weeks (Y.Post.1: *n* = 6; C.Post.1: *n* = 6) and 1 month (Y.Post.2: *n* = 6; C.Post.2: *n* = 6) postpartum. In particular, the second sampling time was ~1–2 weeks postpartum (on average 10 days postpartum), since the exact time of calving cannot be accurately determined. Briefly, the swab samples were collected from the udder skin by rubbing the moistened sterile cotton swab over at least 30 s. When the sample was collected, the swab was immediately placed inside the sampling tube and immersed in Amies medium (1.5 ml) (Universal Transport Medium for Bacterium, Beijing, China). All samples were immediately frozen using liquid nitrogen, transported to the laboratory, and stored at −80°C prior to DNA extraction.

In particular, a total of 24 milk samples from yak and cattle were collected, of which colostrum samples (yak, *n* = 6; cattle, *n* = 6) were collected within 24 h after parturition, and normal milk (yak, *n* = 6; cattle, *n* = 6) was collected at the second week after parturition. To collect milk samples, the udder end was cleaned and disinfected using a cotton gauze pad moistened in 70% ethanol. Approximately, 100 ml of sample from each animal was collected into sterile sampling tubes and kept in an ice box, transported to the laboratory, and stored at −80°C prior to analysis.

### Composition Analysis of Whey Protein in Colostrum and Normal Milk

The whey proteins were extracted from yak or cattle colostrum and normal milk, and the concentration of total whey proteins (TWP), immunoglobulin G (IgG), lactoferrin (LF), α-lactalbumin (α-Lac), β-lactoglobulins (β-Lg), and bovine serum albumin (BSA) was detected by using ELISA method. Bovine standard ELISA kits for IgG, LF, β-Lg, α-Lac, and BSA were purchased from Shanghai MLB 10 Biotechnology Co. Ltd (Shanghai, China). A Coomassie Bradford protein assay kit and BSA standards for total protein analysis were purchased from Sangon Biotech (Shanghai, China).

### DNA Extraction and Illumina Sequencing of 16S RRNA Genes

These 36 samples were applied to the same sample preparation and DNA isolation procedure. Sample preparation was performed prior to DNA isolation to optimize microbial loads for 16S rRNA gene PCR amplification. The sample preparation for each sample group was as follows. The swab samples were oscillated for 15 s to release the bacteria from the swabs, and then centrifuged at 12,000 × g for 10 min at 4°C. After that, the cotton swab was carefully removed, and the centrifugation step was repeated. The obtained pellet was utilized for DNA isolation. Total genomic DNA from all the samples (*n* = 36) was extracted using hexa-decyl tri-methyl ammonium bromide (CTAB) method (18). DNA concentration and purity were monitored on 1% agarose gels. DNA was diluted to a final concentration of 1 ng/μl using sterile distilled water. The bacterial V4 region of the 16S rRNA gene was amplified using F515/R806 universal primers under the following conditions: initial denaturation at 98°C for 1 min, followed by 30 cycles of denaturation at 98°C for 10 s, annealing at 50°C for 30 s, elongation at 72°C for 30 s, and completed by a final extension at 72°C for 5 min. Amplicons were purified using Qiagen Gel Extraction Kit (Qiagen, Germany). Sequencing libraries were generated using TruSeq® DNA PCR-Free Sample Preparation Kit (Illumina, USA) following the manufacturer's recommendations, and index codes were added. The library quality was assessed on the Qubit@ 2.0 Fluorometer (Thermo Scientific) and Agilent Bioanalyzer 2100 system. Finally, the library was sequenced on an Illumina NovaSeq PE250 platform, and 250 bp paired-end reads were generated (Novogene, Tianjin, China).

### Bioinformatics and Statistical Analysis

Paired-end reads were assigned to samples based on their unique barcode and then merged using FLASH (Version 1.2.7, http://ccb.jhu.edu/software/FLASH/) (19). Quality filtering of the raw tags was performed under specific filtering conditions to obtain the high-quality clean tags (20) according to QIIME (Version 1.9.1, http://qiime.org/scripts/split_libraries_fastq.html) (21). Sequences with ≥97% similarity were assigned to the same OTUs by Uparse software (Version 7.0.1001, http://drive5.com/uparse/) (22). A representative sequence for each OTU was screened for further annotation. For each representative sequence, the Silva Database (http://www.arb-silva.de/) (23) was used based on Mothur (Version 1.36.0) algorithm to annotate taxonomic information. Alpha diversity was analyzed to check the complexity of species diversity through observed species richness and Shannon diversity index using QIIME and displayed with R software (Version 2.15.3). For beta-diversity, beta_diversity.py in QIIME was used to obtain distance matrices, and non-metric multidimensional scaling (NMDS) plots of the Bray-Curtis metric were calculated using square root transformed data and visualized in R (vegan package). Permutational multivariate analysis of variance (PERMANOVA) was used to examine the differences in the microbial communities of the udder skin between yak and cattle in different perinatal periods. The linear discriminant analysis (LDA) effect size (LEfSe) algorithm was used for differential analysis to identify significantly different taxa (24). Moreover, intersections between sets of OTUs were visualized using the UpSet plot [with the R package UpSetR (Version 1.3.3)] (25). In addition, to understand the correlations among different core genera, we constructed a co-occurrence network based on the 16S rRNA gene. The bacterial correlations between yak and cattle samples were analyzed, according to the relative abundance of each genus using Spearman's correlation coefficient to construct the co-occurrence network. The correlation was considered significant when the absolute value of Spearman's rank correlation coefficient was >0.6 and *P*-value was smaller than 0.05. The significantly correlated genera were visualized using Cytoscape version 3.7.1 (http://www.cytoscape.org). Meanwhile, the 16S function prediction was employed to standardize the OTU abundance by PICRUSt (26), which was used to remove the effect of the number of copies of the 16S marker gene in the species genome. The predicted functional contents were summarized at KEGG pathway hierarchy level 2 for interpretation and subsequent analysis. In addition, the box plot was constructed to show the differences between the 10 most important potential functions of the microbial community in the udder skin of yak and cattle in different perinatal periods. The Kruskal–Wallis non-parametric test was used to examine the differences among the groups.

### Data Availability

Sequencing datasets of this study are available at the Sequence Read Archive of the National Center for Biotechnology Information under the accession number PRJNA724917 (http://www.ncbi.nlm.nih.gov/bioproject/724917).

## Results

### Analysis of Whey Protein Components in Colostrum and Normal Milk of Yak and Cattle

Through the analysis of the main components of whey protein in milk samples of yak and cattle during the first and second weeks of postpartum, the average contents of TWP, IgG, and LF in the colostrum of yak and cattle were significantly higher than in normal milk (*P* < 0.05, Table 1). In addition, there were significant differences in TWP and IgG of whey protein in colostrum between yak and cattle, among which the TWP and IgG content of whey protein in yak colostrum was significantly higher than those found in cattle (*P* < 0.05). Nevertheless, there was no significant difference in the average contents of β-Lg and α-Lac in colostrum and normal milk between yak and cattle.

**Table 1:** Analysis on the concentration difference of major and minor proteins in colostrum and normal milk between yak and cattle.

**Item**	**Colostrum**		**Normal milk**		**SEM**	***P*** **-value**	
	**Yak**	**Cattle**	**Yak**	**Cattle**		**Yak vs. cattle**	**Colostrum vs. normal milk**
TWP (mg/mL)	78.39^a^	70.19^b^	9.11^c^	7.36^c^	6.94	0.0015	<0.001
IgG (mg/mL)	60.68^a^	53.66^b^	0.78^c^	0.55^c^	5.94	0.0059	<0.001
LF (mg/mL)	1.36^b^	1.61^a^	0.38^c^	0.27^c^	0.13	0.41	<0.001
β-Lg (mg/mL)	2.06	2.07	2.02	1.96	0.04	0.68	0.58
α-Lac (mg/mL)	0.74	0.72	0.7	0.64	0.02	0.26	0.17
BSA (mg/mL)	0.40^b^	0.58^a^	0.38^b^	0.36^b^	0.03	0.07	0.0083

*SEM, standard error of the mean*.

*^a, b, c^Significant levels of major components in whey between yak and cattle (P < 0.05)*.

### Abundance and Diversity of Udder Skin Microbiota of Yak and Cattle During Perinatal Period

**Figure 2:**
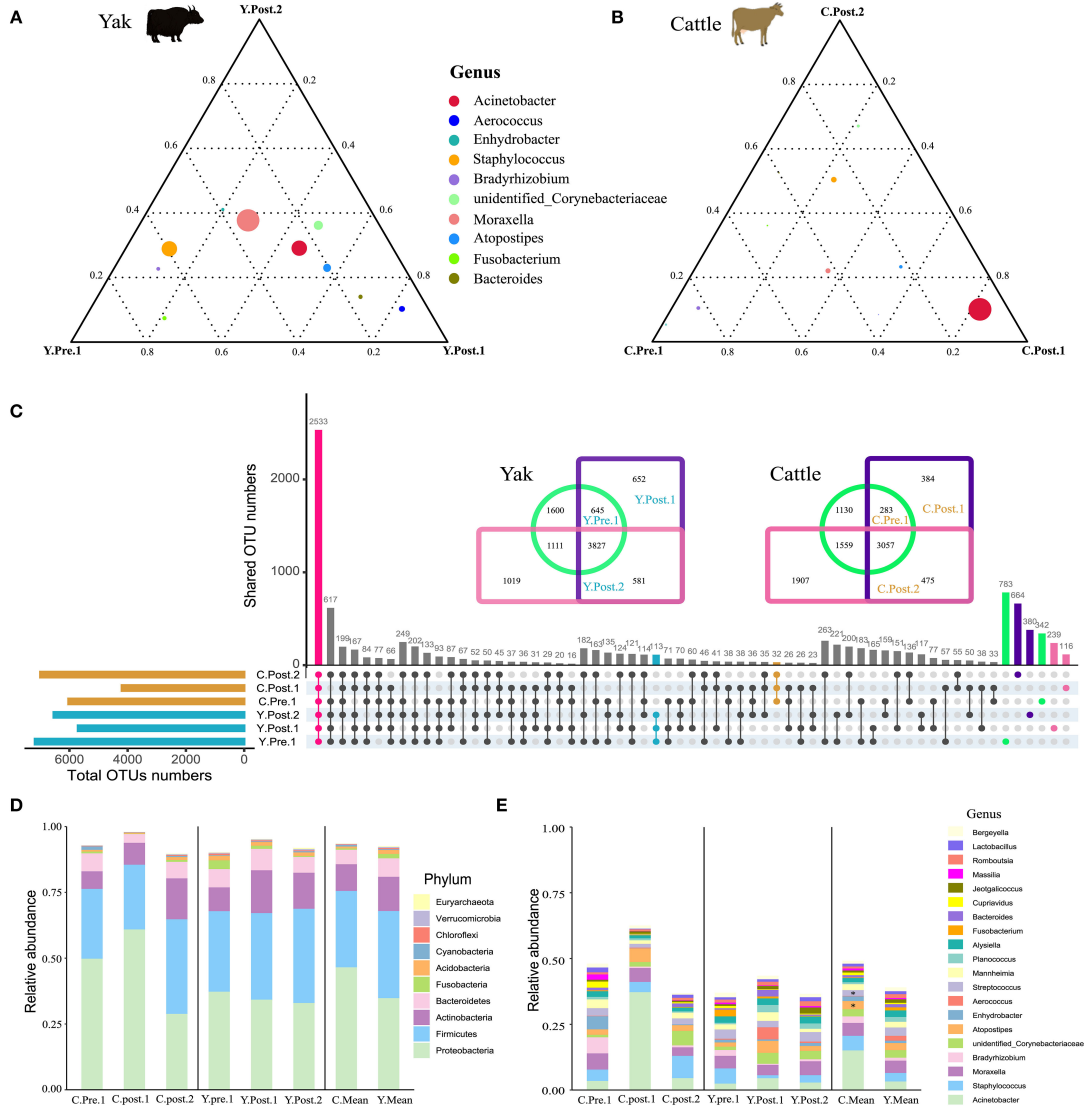
Structure analysis of the core microbial community in udder skin of yak and cattle. The ternary plot command in the vcd package of R software was used to plot the ternary plot at the genus level (top 10) of udder skin microbiota of yak **(A)** and cattle **(B)** at different periods during the perinatal period. The three vertices in the figure represent the sample groups of three different time periods, and the circle represents the species. The size of the circle is proportional to the relative abundance. The closer the circle is to the vertex, the higher the content of the object in this group. **(C)** In order to find the OTU structure of the core microbiota of bovine udder skin, the OTU sharing of all the udder skin microbiota of yak and cattle in different periods during the perinatal period was plotted by upset command of UpSetR package of R software. Using jvenn online (27), OTU concentrations of microbiota in the skin of the respective udders of perinatal yak and cattle were mapped. The bar chart shows the composition of the core microbial community at **(D)** phylum and **(E)** genus levels in the skin of the udder of cattle (left), yak (middle), and mean values of both breeds (right). Significant changes are represented by an asterisk (*T*-test, *p < 0.05).

To explore the dynamic changes of udder skin microbial communities in perinatal cattle, we collected swab samples of udder skin surface from naturally grazed yak (*n* =18) and cattle (*n* =18) at different perinatal periods (Figure 1A). We obtained 2,930,796 quality-filtered 16S rRNA gene sequences from all DNA samples with an average of 81,411 ± 9,621 (mean ± SD) reads per sample. The rarefaction curve and species accumulation box plot of samples reached the plateau (Supplementary Figure S1). A total of 11,077 OTUs were annotated based on 97% sequence similarity, of which the proportion of sequences annotated at the genus levels was 4,016 OTUs (36.26%). We identified 113 unique OTUs in yak and 32 unique OTUs in cattle, while the shared OTUs in yak and cattle during the perinatal period were 2,533 (Figure 2C).

In particular, the observed species richness and Shannon diversity index in the udder skin of yak and cattle within 1–2 weeks after calving were significantly (*P* < 0.05) lower than in the samples obtained before and 1 month after calving (Figures 1B,C). No significant difference was observed in species richness and Shannon diversity index of the udder skin microbial community between yak and cattle within 1–2 weeks of prenatal period and 1 month after postpartum. Moreover, the diversity and richness of microbial community in the udder skin of cattle were significantly lower than those of yak in the same period (*P* < 0.05; Figures 1B,C and Supplementary Table S1).

Next, based on the NMDS analysis of the Bray-Curtis distance matrix, we sought to examine how the microbial community in bovine udder skin varied during different perinatal periods (Figures 1D,E and Supplementary Table S2). We found that the microbial community of the udder skin between yak and cattle was clustered together except during 1–2 weeks postpartum, but there were significant differences in the udder skin microbiota structure of cattle at different perinatal periods [PERMANOVA, *F*_(1, 34)_ = 2.281, *P* = 0.001; Stress = 0.158). Specifically, the microbial community structure of bovine mammary skin in 1–2 weeks postpartum was more dispersed than that of yak in the same period [PERMANOVA, *F*_(1, 10)_ = 3.661, *P* = 0.001; Supplementary Table S2].

### Composition of Udder Skin Core Microbiota of Yak and Cattle During Perinatal Period

Out of the 29 total identified bacterial phyla, four phyla dominated the udder skin microbiota (average cumulative abundance = 93.56%): Proteobacteria (Y: 36.09%; C: 47.79%), Firmicutes (Y: 34.15%; C: 30.18%), Actinobacteria (Y: 13.58%; C: 10.92%), and Bacteroidetes (Y: 8.18%; C: 6.21) (Supplementary Figure S2A). In addition, two archaeal phyla Euryachaeota (Y: 0.24%; C: 0.09%) and Thaumarchaeota (Y: 0.20%; C: 0.06%) were also found in udder skin microbiota (Supplementary Figure S2A). Specifically, within 1–2 weeks after calving, *Acinetobacter* (average abundance increased from 3.45% at prenatal to 37.31% at postpartum) quickly became the highest average member of udder skin microbial community in cattle, opposite to *Enhydrobacter* (average abundance decreased from 4.98% at prenatal to 0.06% at postpartum) and *Bradyrhizobium* (average abundance decreased from 6.12% at prenatal to 0.54% at postpartum) (Supplementary Figures S2A,B). Compared to cattle, variation in the udder skin microbial community of yak before and after calving was smaller, among which *Aerococcus, Atoposipes, Acinetobacter*, and *Moraxella* were the dominant genera of the udder skin microbiota in yak at 1–2 weeks postpartum (Figures 2A,B).

In order to further analyze the composition of the microbial community of the udder skin microbial community in yak and cattle, we used set analysis to find that 2,533 OTUs were enriched in all OTU sequences of yak and cattle udder skin (Figure 2**C**). These core microbial communities were also composed of Proteobacteria, Firmicutes, Actinobacteria, and Bacteroidetes (Figure 2D and Supplementary Figure S3A). We found dynamic changes in the core microbiota of udder skin in yak and cattle at different stages of the perinatal period, particularly in the cattle at 1–2 weeks postpartum (Figures 2D,E and Supplementary Figure S3). We also found that among all OTU sequences of udder skin, the specific OTUs of yak (113 OTUs) were higher than those of cattle (32 OTUs). In addition, we found that the shared OTU of udder skin microbial community in yak during the perinatal period was 3,827 while that in cattle was 3,057 (Figure 2C).

### Effects of Perinatal Period on Udder Skin Microbial Community of Yak and Cattle

**Figure 3:**
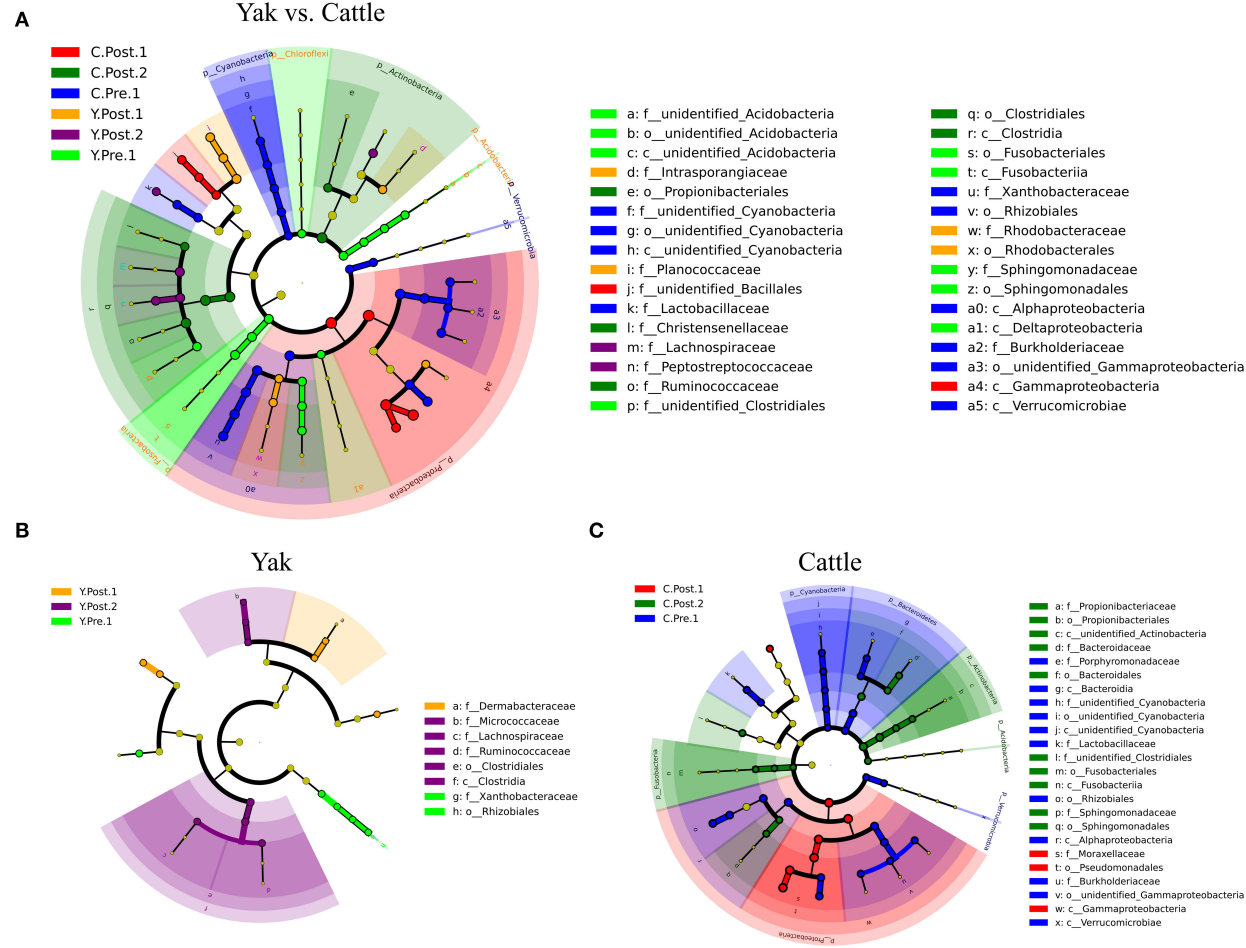
LEfSe analysis of microbial community structure of bovine breast skin during the perinatal period. **(A–C)** The cladograms indicate the phylogenetic distribution of the udder skin microbiota of yak and cattle during different periods of the perinatal period using the linear discriminant analysis (LDA) effect size (LEfSe) method. The diameters of circles are proportional to the abundance of a taxon. Circles represent taxonomic ranks from domain to species levels from inside to out layers. The LDA cut-off score is 3.5. Letters in front of OTUs represent taxonomic levels (p, phylum; c, class; o, order; f, family).

Consistent with the microbial composition results (Supplementary Figure S3), there were obvious similarities between the udder skin microbiota of yak and cattle (Figure 3). For the yak udder skin microbiota in the perinatal period, *Staphylococcus* and *Bradyrhizobium* were the dominant genera at prepartum, but *Dermabacteraceae* increased to become the dominant family within 1–2 weeks postpartum, and *Ruminococcaceae, Lachnospiraceae*, and *Micrococcaceae* were dominant bacterial families after 1 month postpartum (Figure 3B and Supplementary Figure S4B). At 1–2 weeks postpartum, Proteobacteria quickly became the dominant phylum of the udder skin microbiota of cattle, of which *Acinetobacter* was the dominant genus (Figure 3C and Supplementary Figure S4C). However, Actinobacteria and Fusobacteria were relatively abundant after 1 month postpartum (Figure 3C). Moreover, the LEfSe results found that the udder skin microbiota of cattle was mainly enriched in Cyanobacteria, Proteobacteria, and Actinobacteria, while that of the yak was mainly enriched in Fusobacteria and Acidobacteria (at the phylum level, Figure 3A and Supplementary Figure S4).

### Co-Occurrence Network Analysis of Udder Skin Core Microbiota of Yak and Cattle

**Figure 4:**
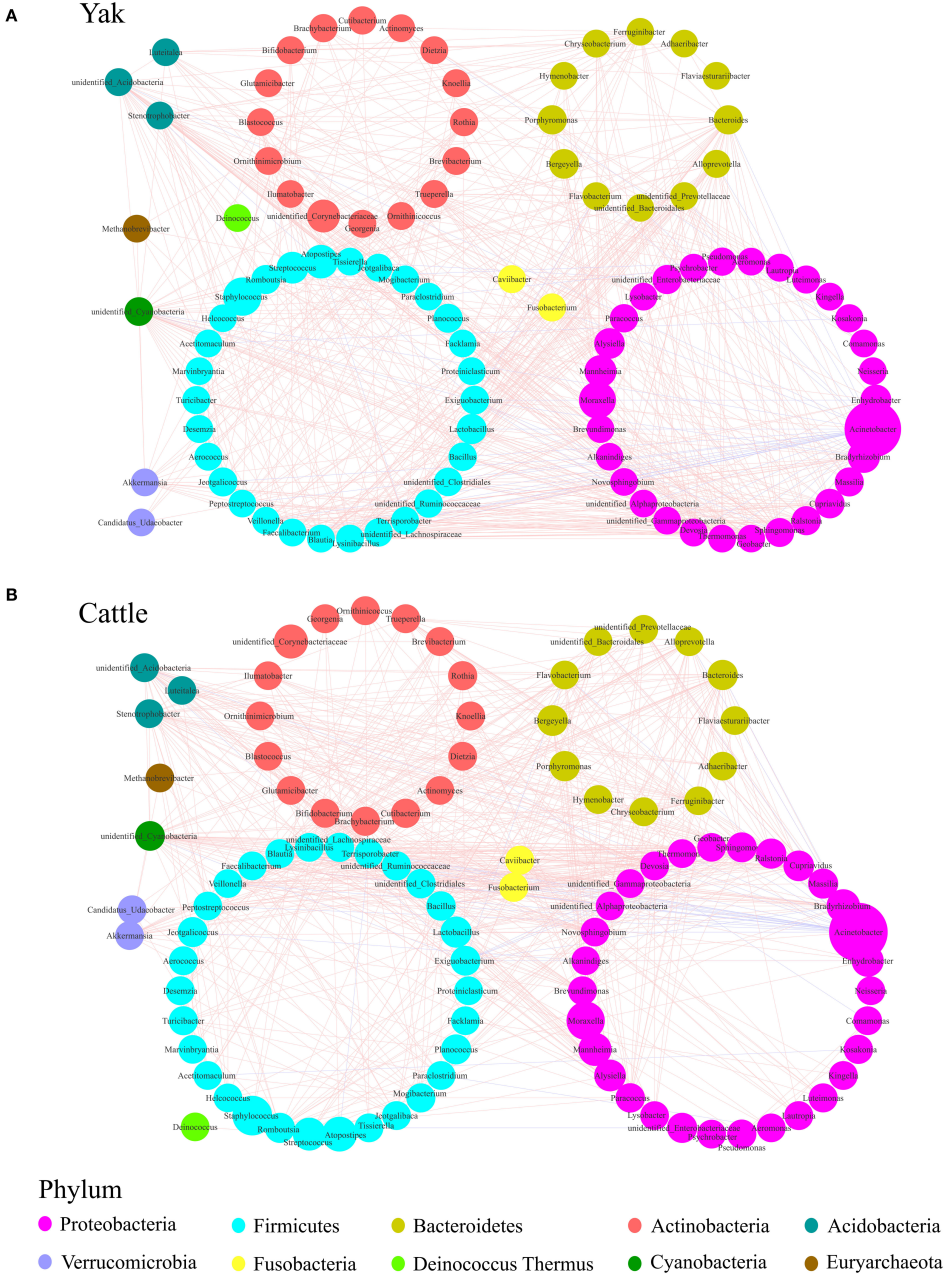
Genera co-occurrence network of core microbiota of udder skin between yak and cattle based on Spearman's correlation algorithms. Spearman's method was used to analyze the correlation between the udder skin core microbiota of yak **(A)** and cattle **(B)**, respectively, and the Cytoscape_3.7.1 software was used to draw the co-occurrence network of the core microbiota with *P*-value < 0.05 and the absolute value of correlation >0.6. Each node presents a bacterial genus. The node size indicates the relative abundance of each genus per group, and the density of the dashed line represents Spearman's correlation coefficient. Red links stand for positive interactions between nodes, and blue links stand for negative interactions.

To describe the potential relationship between bacteria occurring in the core microbial community of bovine udder skin, we further constructed co-occurrence networks of genera from the udder skin core microbiota of yak and cattle based on Spearman's correlation coefficients (Supplementary Figure S5 and Supplementary Table S3). We identified that the dominant bacterial genera in both the networks were mainly distributed from five major phyla, such as Firmicutes, Proteobacteria, Actinobacteria, Bacteroidetes, and Fusobacteria (Supplementary Figure S5). In particular, a strong positive correlation among genera was observed in the co-occurrence network of yak (Figure 4A). In contrast, the co-occurrence network of cattle featured a relatively simple network (Figure 4B). The correlation between the udder skin core microbiota of yak was distinctly increased compared to that of cattle (Supplementary Table S4). Meanwhile, we found that there was a strong correlation between *Acinetobacter, Bacteroides, Stenotrophobacter, Fusobacterium, Brevibacterium, unidentified_Cyanobacteria*, and other microbial genera in the co-occurrence network of udder skin core microbiota in yak and cattle (Figures 4A,B).

### Prediction of Potential Function of Udder Skin Microbial Community of Yak and Cattle

**Figure 5:**
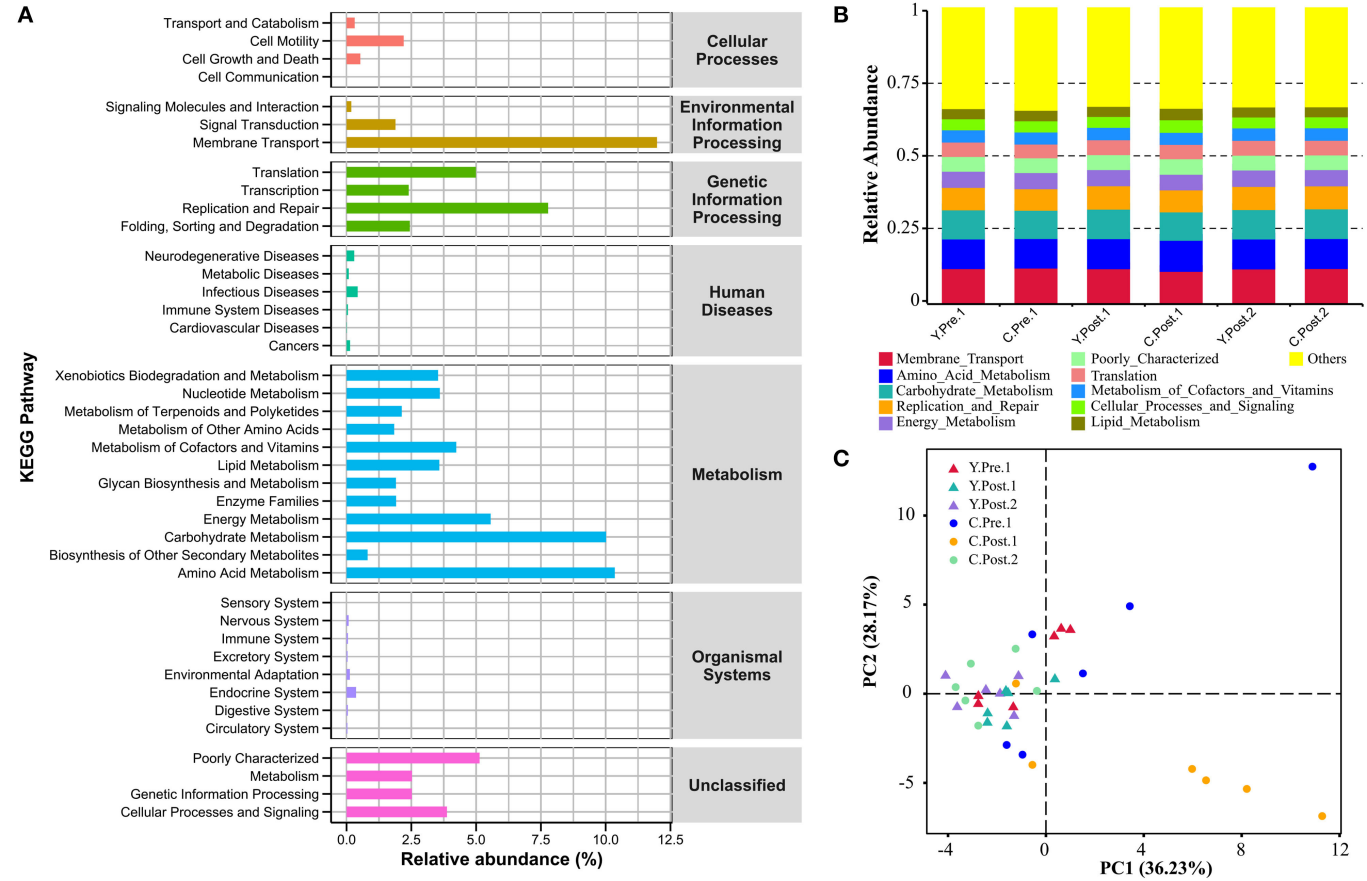
Prediction of the potential function of microbial community in bovine udder skin using PICRUST. **(A)** The bar graph shows the main potential function of the udder skin microbial community of yak and cattle in the perinatal period on KEGG Level 1 and Level 2. **(B)** The stacked bar chart shows the major microbial functions of the microbial community in the udder skin of yak and cattle at KEGG Level 2 at different time points during the perinatal period. **(C)** Principal component analysis (PCA) shows microbial functional diversity across all samples.

In this study, the chosen reference OTUs were used to match the KEGG database to predict microbial functions. Generally, the potential functions of the udder skin microbiota of yak and cattle were mainly manifested in membrane transport (Y: 12.08%; C: 11.89%), amino acid metabolism (Y: 10.30%; C: 10.41%), carbohydrate metabolism (Y: 10.12%; C: 9.91%), replication and repair (Y: 7.92%; C: 7.64%), energy metabolism (Y: 5.60%; C: 5.52%), and poorly characterized mechanism (Y: 5.11%; C: 5.16%) (Figures 5A,B). In addition, we also performed a PCA analysis of the relative abundance of the KEGG pathway to reveal the clustering of the samples (Figure 5C). The results of PCA and box plot both revealed that the perinatal period had little effect on the potential function of the microbial community in the udder skin of yak (Figure 5C and Supplementary Figure S6). However, the perinatal period had an important effect on the potential function of the udder skin microbial community in cattle, particularly within 1–2 weeks of the postnatal period, and the potential function of the udder skin microbiota in carbohydrate metabolism was significantly different from other periods (Kruskal–Wallis, *P* < 0.05; Figure 5C and Supplementary Figure S6).

## Discussion

Mammalian colostrum and milk not only serve as complete nutrient sources for offspring, but also contain a complex array of bioactive molecules capable of modulating intestinal immune homeostasis of newborns, preparing for a microbe-rich extrauterine environment (5, 28). Immunoglobulin, antibacterial peptide, lysozyme, lactoferrin, and oligosaccharide are some of the immunomodulatory components of milk (29–31) that can target and inactivate pathogens (32). However, we found that the TWP and IgG of whey protein were significantly higher in yak colostrum than in cattle colostrum, which may help yak calves to better adapt to the extreme natural environment of the QTP. Compared with dairy cow milk, the fat content of yak milk is almost twice that of dairy cow (*Bos taurus*) milk (15), but its yield is only about 10% of the dairy cow (6). In addition to supporting the immature innate immune response of newborns, these immunomodulatory compounds may also act as an important part of the defense mechanism of the udder itself and protect it against intra-mammary infections by pathogenic and opportunistic microorganisms (5, 33). Therefore, we used high-throughput sequencing of 16S rRNA genes to investigate the dynamics of microbial community structure in bovine udder skin during the periparturient period. We found that 1–2 weeks postpartum had a significant effect on the structure and stability of the microbial community in the udder skin of yak and cattle, in which the richness and diversity of the microbial community were significantly reduced. The defense mechanisms of the udder against microbial colonization are modulated by several host-associated and environmental factors (5, 34). This may be due to the fact that the nutrient supply and dry matter intake of perinatal cows are insufficient to meet the energy demands for body maintenance, colostrogenesis, and milk production (35). This unavoidable state of negative energy balance after calving can result in the development of several metabolic disorders that could impair the immune system and subsequently cause alterations in the skin microbiota (36). Similarly, studies have shown that high-yielding cows may be at increased risk of mastitis due to changes in housing, hygiene, and feeding conditions in the period around calving (37), and the highest peak of new intramammary infections is usually recorded in the first 2–3 weeks after calving (38). We also found that the structure of the microbial community in the udder skin of periparturient yak was less variable than that of cattle, which may be related to the adaptation of yak to the extreme natural environment on the QTP during the long-term natural selection. Additionally, genetic, physiological, and environmental factors are capable of modulating the defense mechanisms of the bovine mammary gland against each of these pathogens (39, 40). However, within complex ecosystems, certain species play disproportionately large roles in shaping the overall structure and stability of the community (41).

To date, a combination of culture-dependent and DNA-based approaches has been used to explore the diversity of bacterial communities colonizing the teat apex of dairy cows (42, 43). These studies have revealed wide diversity in the occurrence of the commensal, pathogen, and skin-associated opportunistic bacteria from four major bacterial phyla, namely Actinobacteria, Bacteroidetes, Firmicutes, and Proteobacteria, and can reside on the skin of the teat apex of dairy cows. Similarly, the results of our study also showed that the microbial community in the udder skin of yak and cattle was mainly composed of Proteobacteria, Firmicutes, Actinobacteria, and Bacteroidetes. Besides, archaea members were found in the microbial community of bovine udder skin, such as Euryachaeota and Thaumarchaeota. The most commonly identified genera include *Acinetobacter, Moraxella, Staphylococcus, unidentified_Corynebacteriaceae, Bradyrhizobium, Lactobacillus, Atopostipes, Alysiella*, and *Fusobacterium*. *Staphylococcus, Ruminococcaceae, Bacteroidales, Clostridiales*, and *Pseudomonas* have also been identified as predominant constituents of healthy colostrum microbiota (44). *Staphylococcus chromogenes*, followed by *Staphylococcus simulans, Staphylococcus xylosus, Staphylococcus haemolyticus*, and *Staphylococcus epidermidis*, are the *Non-aureus staphylococci* (NAS) species most frequently isolated from cow milk (45, 46). Some studies found that the ability of some NAS species (e.g., *S. chromogenes*) to produce a wide range of bacteriocins capable to inhibit the growth of major mastitis pathogens is a good example of mechanisms by which commensal microbiota may contribute to the modulation of mastitis susceptibility (47, 48). Other than NAS, *Acinetobacter, Aerococcus*, and *Corynebacterium* are among the most frequently identified genera on the skin of teat apices in bovine (49). In our study, *Acinetobacter* quickly became the most abundant genus in the microbial community in the udder skin of cattle at 1–2 weeks after parturition, which indicates that there might be a potential risk of mastitis in cattle due to the imbalance of microbiota stability in the udder skin during this period. Additionally, this finding also suggests that microbiota present on the cattle skin may be more susceptible to common environmental stress factors such as housing, hygiene, and feeding conditions during the perinatal period (37).

The composition, stability, and function of the microbial community in the skin are driven by the interaction between host factors and microbiota (50). We found that the changes in the microbial community structure of yak udder skin were different from those of cattle during the perinatal period, particularly the differences in the cattle were the most significant. The feeding conditions (indoor and pasture) of dairy cows play a central role in the formation of the mammary microbiome. For example, the microbial diversity of udder skin samples is higher during the grazing season than during the feeding season (51). Additionally, some studies have reported that commensal microbiota that inhabits various niches of the udder, including teat apex, teat canal, and intramammary ecosystem, can modulate the susceptibility of a cow to intramammary infection by mastitis pathogens via direct microbe–microbe cross-talk, indirect stimulation of immunity, or both (5, 40, 52). We also found that there was a strong positive correlation among Proteobacteria, Firmicutes, Actinobacteria, and Bacteroidetes, and the understanding of the interaction among these core bacteria can provide a reference for further screening of the minimal core drive bacteria as probiotics. Recent studies have shown that using the ecological network of the community to identify minimum sets of its driver species and controlling these microbial communities may help us restore natural ecosystems and maintain healthy human microbiota (53). In addition to the known dysfunctions in the barrier function of the skin and immunologic disturbances, evidence is rising that frequent skin disorders, such as atopic dermatitis, might be associated with the disorders of the microbial community and changes in the skin microbiome (54, 55). We found that the perinatal period has an important effect on the structure and potential function of the udder skin microbiota of yak and cattle; in particular, the potential function of the udder skin microbiota of yak and cattle during 1–2 weeks postpartum is significantly different from other periods in carbohydrate metabolism. Compared to the late lactation stage, calves mainly feed on breast milk in the early stage, resulting in a large amount of milk attached to the udder skin surface of cattle, which increases the function of the udder skin microbiota mainly focusing on carbohydrate metabolism. Some studies found that the ability of some NAS species to produce a wide range of bacteriocins, capable of inhibiting the growth of major mastitis pathogens, is a good example of mechanisms by which commensal microbiota may contribute to the modulation of mastitis susceptibility (47, 48). However, this study is only a preliminary investigation of the microbial community dynamics in the udder skin of healthy yak and cattle during the perinatal period in the same habitat, and the next step is to delve deeper into the differences in the microbiota structure and function of the udder skin between healthy and mastitis yak. In addition, we found several deficiencies in our study, such as a small number of animals, a lack of diagnosis for mastitis, and a lack of controls for the DNA extraction kits, PCR amplification, and swab cultures. Although functional prediction using the 16S rRNA genome may provide preliminary information for studies on the udder skin microbial functions in bovines during the perinatal period, the detailed functions of the udder skin microbiota need to be further determined by advanced techniques, such as metagenomics, metatranscriptomics, and metabolomics.

## Conclusion

The present study revealed that the perinatal period has important effects on the composition and stability of bovine udder skin microbiota. Compared to the cattle in the same habitat, the variation of microbial community structure and diversity of udder skin of yak during the perinatal period was smaller. Although yak and cattle share a similar udder skin core microbiota, the relative abundance of *Acinetobacter* in the udder skin of cattle during 1–2 weeks postpartum rapidly increased to become the dominant genus, and the average relative abundance of *Atopostipes* and *Streptococcus* in the udder skin of cattle was significantly higher than that of yak.

## Data Availability Statement

The datasets presented in this study can be found in online repositories. The names of the repository/repositories and accession number(s) can be found in the article/Supplementary Material.

## Ethics Statement

All procedures involved in animals care and their use were in strict accordance with the guide-lines for the Care and Use of Laboratory Animals, Lanzhou Institute of Husbandry and Pharmaceutical Sciences, CAAS, China (SYXK-2019-0012). Written informed consent was obtained from the owners for the participation of their animals in this study.

## Author Contributions

JZha and XD designed the study, interpreted the data, and wrote the manuscript. JZha, AA, RD, ZL, MD, IB, and JZhe collected the samples. JZha, AA, and YJ performed bioinformatics and statistical analyses. PY, GS, and XD guided the data analysis and revised the manuscript. All authors have read and approved the final manuscript.

## Funding

This work was supported by a grant from the Innovation Program of the Chinese Academy of Agricultural Sciences (25-LZIHPS-04) and the International Cooperation and Exchange Program of the National Natural Science Foundation of China (31461143020).

## Conflict of Interest

The authors declare that the research was conducted in the absence of any commercial or financial relationships that could be construed as a potential conflict of interest.

## Publisher's Note

All claims expressed in this article are solely those of the authors and do not necessarily represent those of their affiliated organizations, or those of the publisher, the editors and the reviewers. Any product that may be evaluated in this article, or claim that may be made by its manufacturer, is not guaranteed or endorsed by the publisher.
