# Supplementary material for: Comparison of Dynamics of Udder Skin Microbiota From Grazing Yak and Cattle During the Perinatal Period on the Qinghai–Tibetan Plateau
Source: Front Vet Sci. 2022 May 27;9:864057. doi: 10.3389/fvets.2022.864057 (PMC9187117; doi:10.3389/fvets.2022.864057)
Supplement: Supplementary file 5 [file Data_Sheet_1.docx]

**Supplementary Material**

**Table Legends**

**Table S1.** Beta diversity of microbial communities in the udder skin of periparturient yak and cattle (ADONIS)

**Table S2.** Correlation Analysis of udder skin core microbiota of yak and cattle at perinatal period

**Table S3.** Correlation Analysis of udder skin core microbiota between yak and cattle at perinatal period

**Figure Legends**


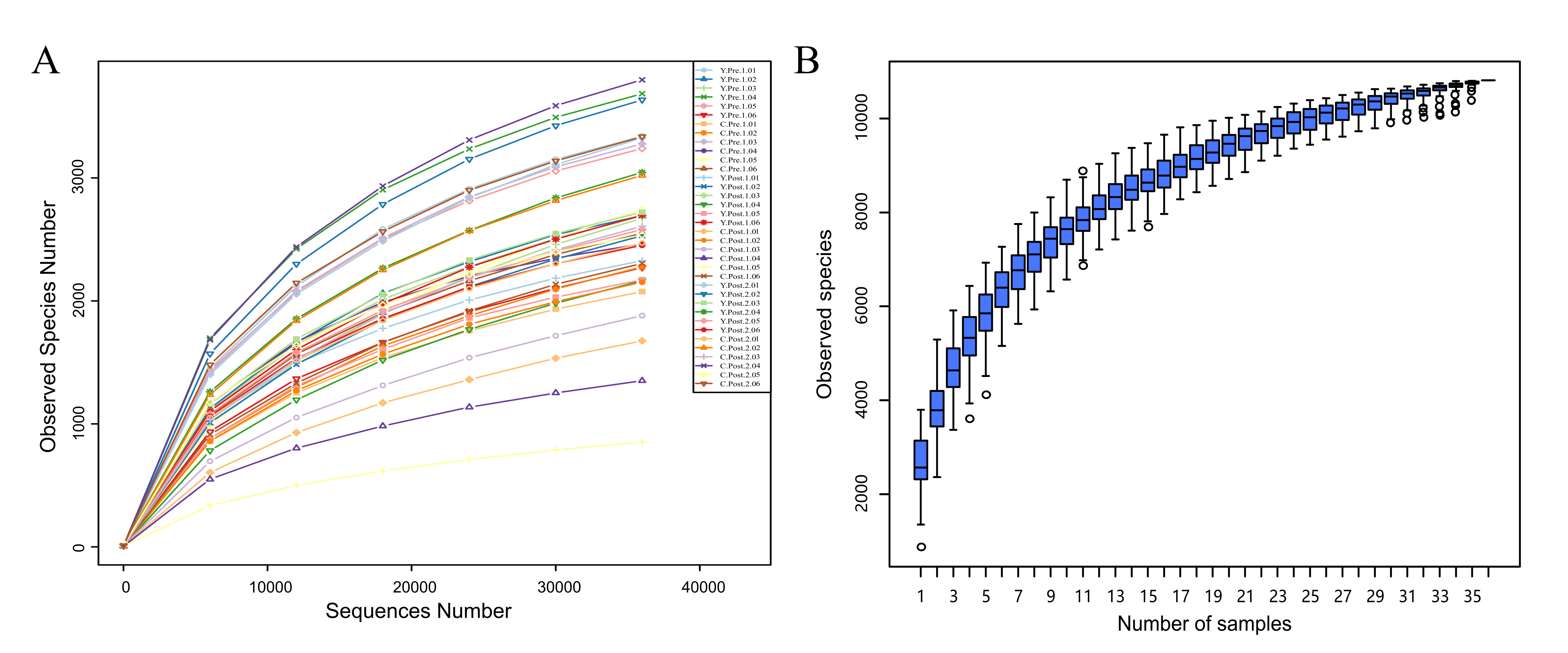


**Figure S1.** Diversity analysis of microbial community in bovine udder skin during perinatal period. (A) Rarefaction curve for each sample. (B) Species accumulation boxplot for each sample.


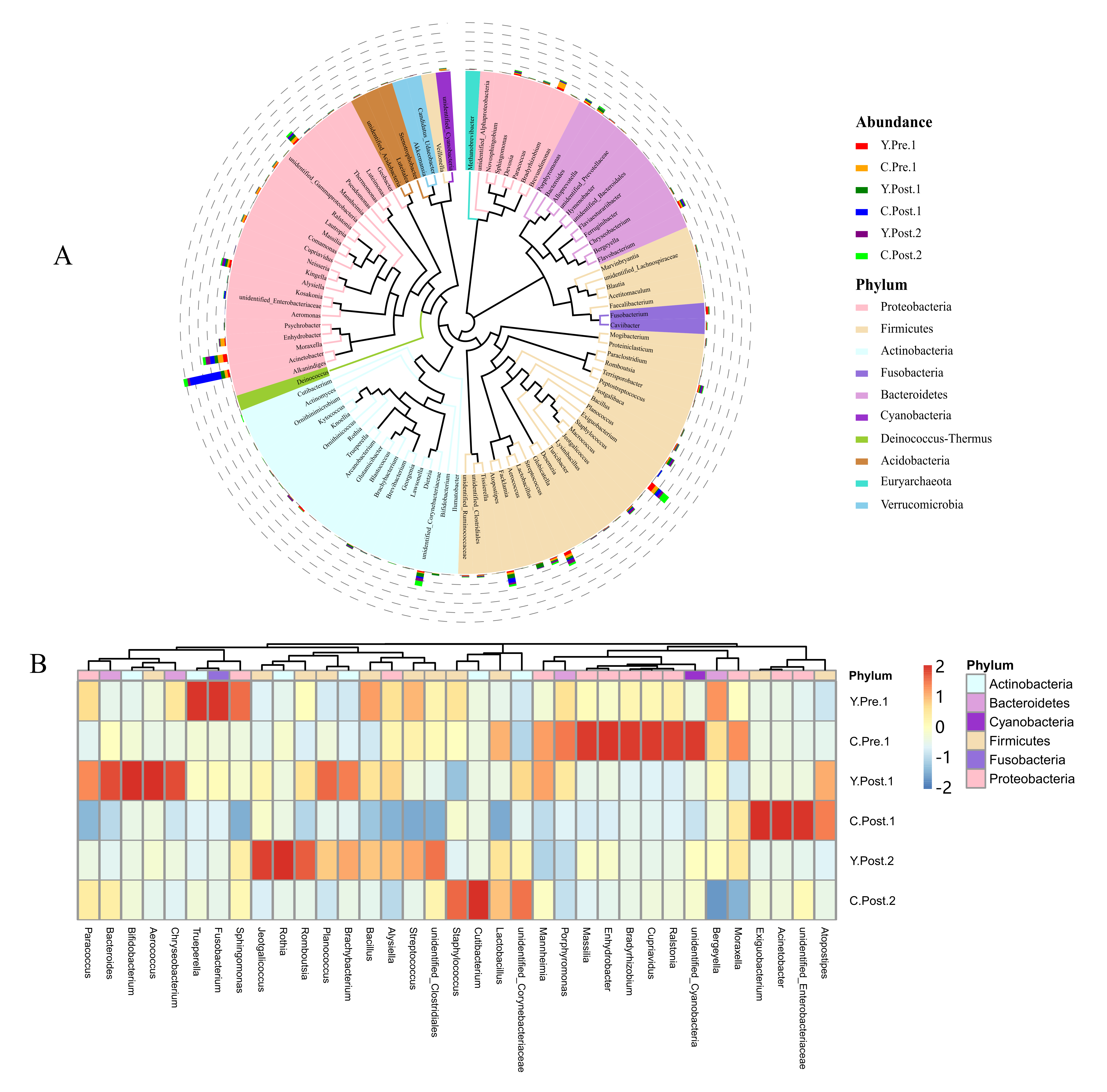


**Figure S2**. Microbial community composition of udder skin of yak and cattle in periparturient. (A) Circos plot. The microbial communities of different samples are phylogenetic at the genus level (top 100). The color of branches and fans indicated the corresponding phyla, and the column graph on the outside of the fan ring indicated the abundance distribution of the genus in different samples. (B) Clustering heat map of species abundance. According to the species annotation and abundance information of all samples at the genus level, the top 35 genera with abundance were selected to draw the cluster heat map of species abundance, in which the is the sample information and the longitudinal is the species annotation information. The cluster tree on the upper side of the map is a species cluster tree.


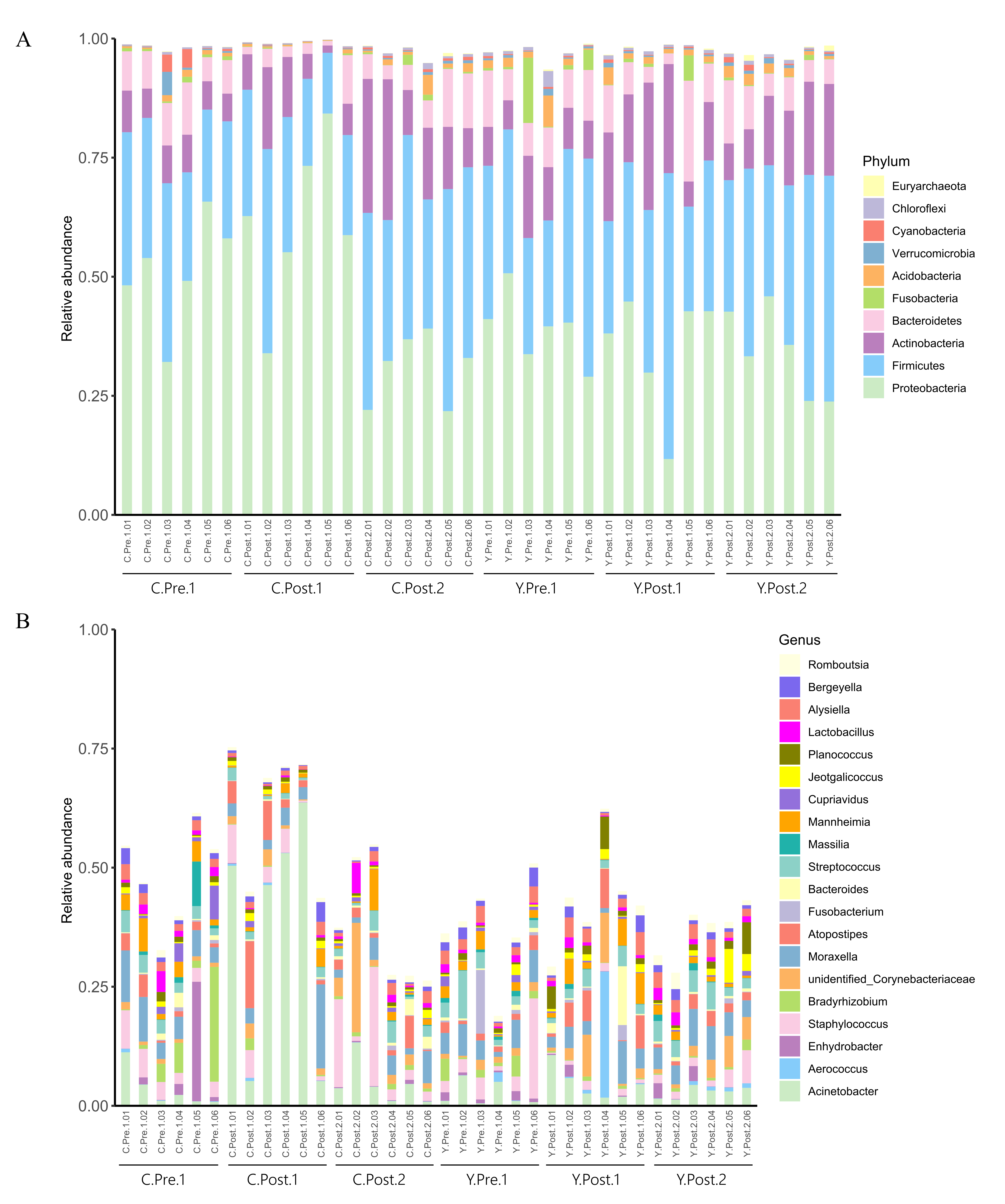


**Figure S3**. The core microbial community composition of udder skin of yak and cattle in different periods during perinatal period. Bar chart showing the composition of the core microbial community at (A) phylum and (B) genus levels in the udder skin of cattle (left) and yak (right).


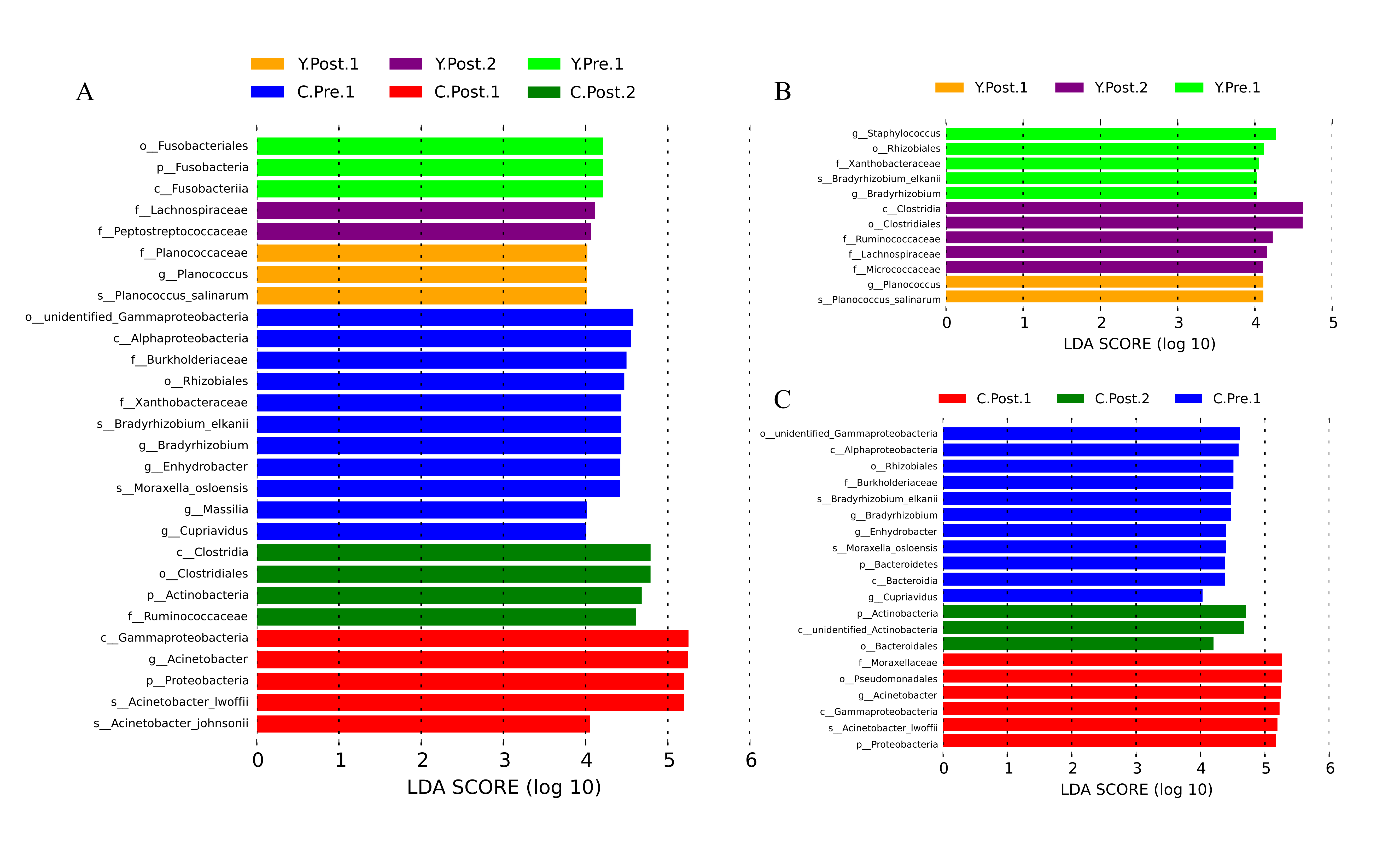


**Figure S4.** LEfSe analysis of microbial community structure of bovine breast skin during perinatal period. (A-C) Bar plots showing differential abundant udder skin microbes of yak and cattle at different period during perinatal period, as identified by linear discriminant analysis (LDA) effect size (LEfSe). The bar plot shows scores for all the taxa with a LDA score ≥ 4. Letters in front of OTUs represent taxonomic levels (p, phylum; c, class; o, order; f, family; g, genera; s, species).


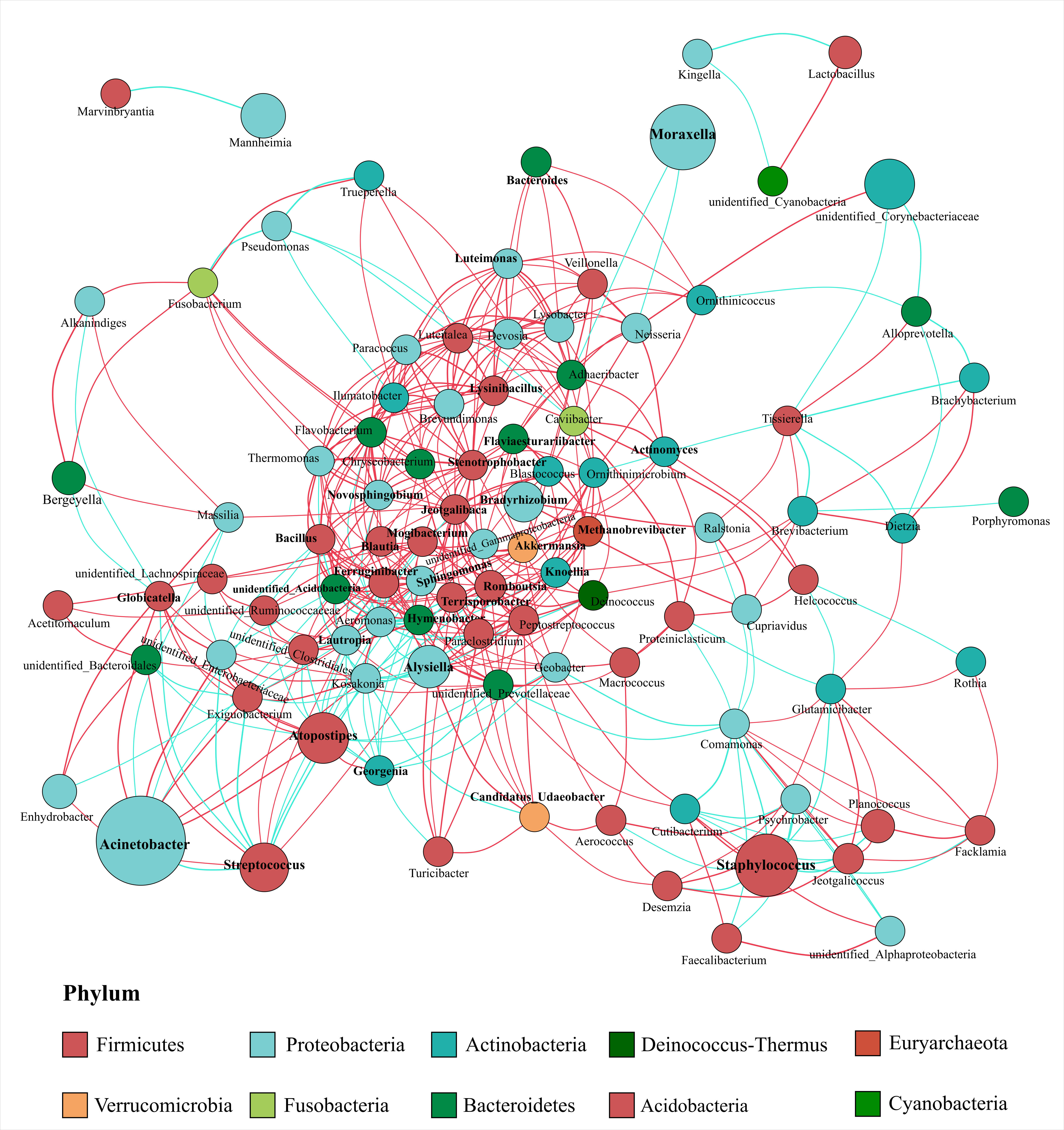


**Figure S5.** Genera co-occurrence network of udder skin core microbiota of yak and cattle based on the Spearman correlation algorithms. The map of the species interaction network analysis; a circle represents a bacteria, the size of the circle represents its relative abundance, different color represents different classification at phylum level, the line between the circles represents the correlation between the two bacteria is significant (P < 0.05), the red color of the line represents a positive correlation, while the blue one represents the negative correlation, the line is more rough, corresponding correlation coefficient value is greater.


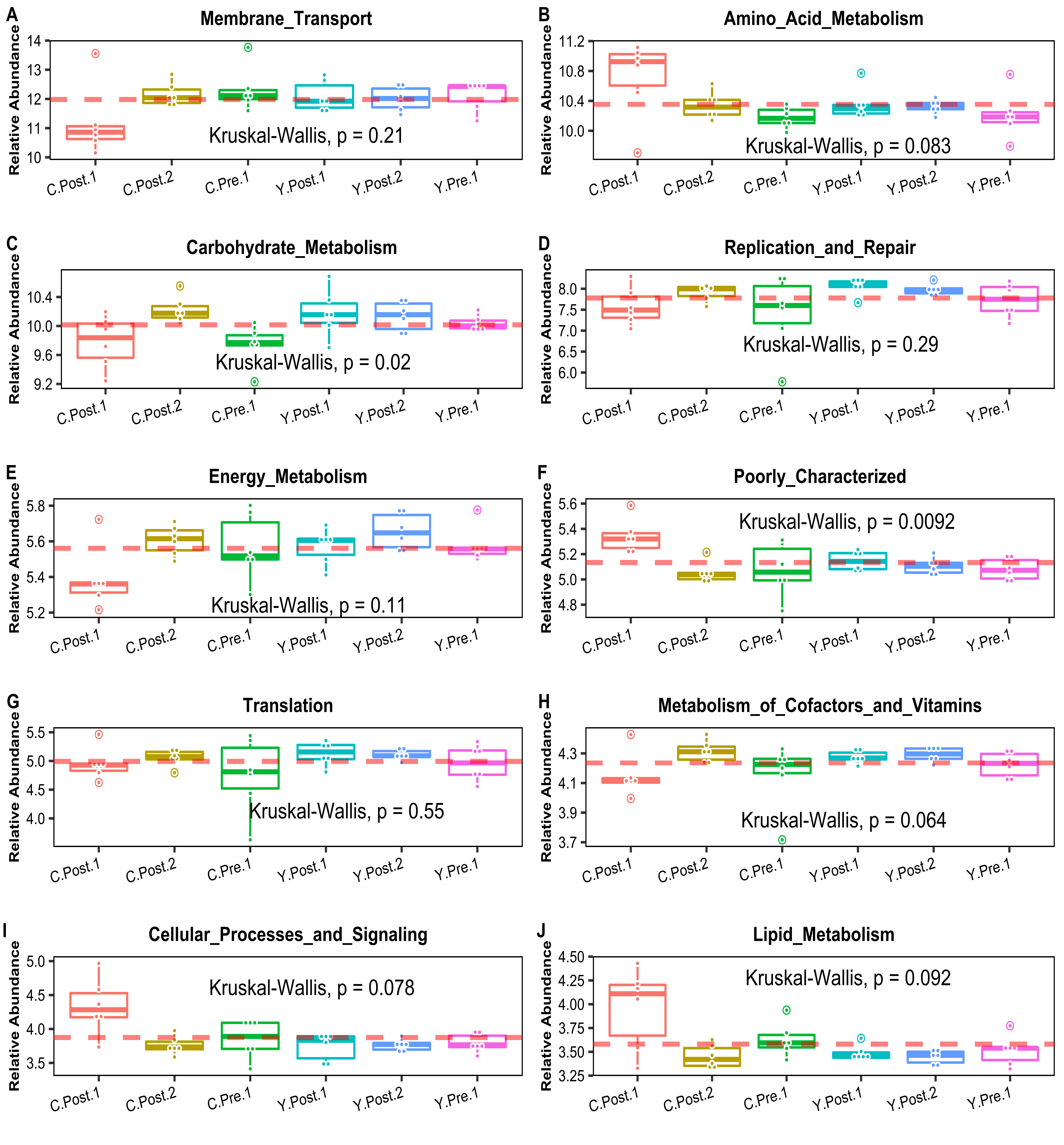


**Figure S6.** The difference of main function of microbiota in bovine udder skin during perinatal period. (A-J) The box plot shows the differences of the 10 most important potential functions of the microbial community in the udder skin of yak and cattle in different perinatal periods. The Kruskal-Wallis non-parametric test was used to examine the differences among the groups.
